# Wenxin Granules Influence the TGF*β*-P38/JNK MAPK Signaling Pathway and Attenuate the Collagen Deposition in the Left Ventricle of Myocardial Infarction Rats

**DOI:** 10.1155/2019/3786024

**Published:** 2019-12-12

**Authors:** Ya Huang, Aiming Wu, Lixia Lou, Dongmei Zhang, Bo Nie, Yizhou Zhao, Keke Liu, Mingjing Zhao, Hongcai Shang

**Affiliations:** Dongzhimen Hospital Affiliated to Beijing University of Chinese Medicine, Key Laboratory of Chinese Internal Medicine of Ministry of Education and Beijing, Beijing 100700, China

## Abstract

**Background:**

A large number of proinflammatory/anti-inflammatory cytokines are produced in the extracellular matrix (ECM) after myocardial infarction (MI), and the inflammatory pathways activated by these inflammatory stimuli are involved in the regulation of lesions with excessive accumulation of ECM. Wenxin granules can play a protective role against MI, but the mechanism of its effect on the inflammatory pathway and ECM collagen expression is still unclear.

**Objective:**

To verify the effect of Wenxin granules on the inflammatory pathway and collagen expression after MI.

**Method:**

The proximal left anterior descending coronary artery in rats was ligated to induce acute MI. Then, animals were randomly assigned to the model group, the Carvedilol group, and the Wenxin Granules group. In addition, sham operation rats were used as the control group. 10 rats were allocated in each group. Gavage was given once a day for 4 weeks. The changes of cardiac hemodynamics were detected by the catheter method, morphological changes were observed by HE staining, and myocardial tissue collagen volume was counted by Immunohistochemistry combined with Masson staining, and the expression of inflammatory TGF*β*-p38/JNK MAPK signal pathway markers was detected by Western blot.

**Results:**

Wenxin granules could significantly improve the hemodynamics, so that the fibrosis scar was relatively dense and uniform, and the residual myocardium was relatively neat, while Collagen type I and III volume and TGF*β* expression levels were lessened. Although there were no differences in the expression of CTGF, p38, and JNK proteins, their phosphorylation levels showed significant differences.

**Conclusion:**

Wenxin granules can affect the inflammation-related TGF*β*-p38/JNK MAPK signaling pathway and change the structural properties of myocardium and scar after MI by attenuated collagen deposition in the left ventricular myocardial tissue to improve cardiac function.

## 1. Introduction

Cardiovascular disease is still the main cause of health loss in all regions of the world, and the disease burden of ischemic heart disease, especially MI, ranks first in this field. In recent years, the importance of inflammation in MI has attracted much attention, and the extracellular matrix (ECM) is an important factor that cannot be ignored in the inflammatory response of MI [1]. The inflammatory response of acute MI plays an important role in determining the size of MI and affecting ventricular remodeling [2]. Many cytokines are produced in the inflammatory repair stage, among which the main one is transformed growth factor-beta (TGF*β*). On the one hand, it acts as an anti-inflammatory medium, preventing inflammatory cells from spreading outward and targeting normal cardiac cells in uninfarcted areas. On the other hand, it acts as an inflammatory stimulant, regulating changes in the overall microenvironment by activating inflammatory signaling pathways, such as inducing enrichment and deposition of extracellular matrix [3]. Dynamic changes in the components of the ECM also play a key role in regulating the cellular response that mediates cardiac repair. The disorder of the ECM homeostasis is an important marker of ventricular remodeling after MI, which is mainly manifested as collagen deposition, showing the characteristics of cardiac fibrosis. Structural cardiac remodeling leads to irreversible heart failure, and the loss of cardiac function is closely related to the degree of myocardial fibrosis.

In clinical practice, PCI and other surgical methods are usually used for revascularization, but postoperative reperfusion injury can aggravate the proinflammatory response, and surgery cannot prevent ischemia-induced progressive myocardial fibrosis [4]. Currently, there is no effective chemical intervention for myocardial cell injury, especially for the pathological changes of the ECM. Under the circumstances, many studies have focused on improving the heart's inherent abilities to cope with acute injury by activating some growth factors, expecting to regulate the benign repair of the heart by intervening in the pathophysiological mechanism of myocardial autoreactivity, leading to better clinical outcomes [5]. The influence of signaling pathways activated in inflammatory response on ventricular remodeling is one of the research hotspots in recent years. Inflammatory cytokines are produced in large quantities in the ECM. In turn, inflammatory signaling pathways have a significant influence on the changes in ECM components. ECM deposition is mainly the deposition of collagen, which maintains the metabolic balance under physiological conditions while tends to be unbalanced under the influence of inflammation. Too little collagen could not repair the myocardium completely, while collagen deposition aggravated myocardial fibrosis and adverse ventricular remodeling [6, 7]. Therefore, studying the mechanism of changes in the inflammatory pathway and the ECM component may be a potential direction and target for the treatment of MI.

Nonclassical MAPK signaling pathway activated by TGF*β* is a key signaling pathway involved in inflammatory and fibrotic responses. Accumulating evidence shows that the P38/JNK MAPK signaling cascade is involved in the inflammatory response of MI and affects the changes of ECM components, especially collagen, which plays an important regulatory role in ventricular remodeling [5, 8, 9]. The main characteristics of myocardial injury after MI are obstructed blood circulation and insufficient blood and oxygen supply to myocardial tissue. However, Wenxin granule is a Traditional Chinese Medicine (TCM) compound with the main function of improving blood circulation, which happens to be symptomatic treatment. After long-term clinical application and basic experimental studies, Wenxin granule has been proved to have a significant protective effect on ischemic heart disease, but its mechanism is not clear. Previous experimental studies have suggested that Wenxin granules have anti-inflammatory effects and play an inhibitory effect on myocardial fibrosis [10]. Wenxin granules also can interfere with the expression of collagen type I in the myocardium, but the effect on collagen type III expression has not been reported [11]. Whether Wenxin granules can play an anti-ischemic role by regulating the changes of inflammation and the ECM is a question worthy of further study.

## 2. Materials and Methods

### 2.1. Experimental Agents

Wenxin granules were provided by Shandong Buchang Pharmaceuticals Co., Ltd. (national drug approval number: Z10950026). Carvedilol tablets were purchased from Shandong Qilu Pharmaceutical Co., Ltd. (national drug approval number: H20000100). Anti-collagen type I antibody, anti-collagen type III antibody, anti-TGF*β*1 antibody, and anti-CTGF antibody were rabbit anti-rat antibodies purchased from Abcam, American. Phospho-p38 MAPK (Thr180/Tyr182) antibody, p38 MAPK antibody (Thr180/Tyr182) antibody, SAPK/JNK antibody, Phospho-SAPK/JNK (Thr183/Tyr185) antibody were recombinant human fusion protein anti-rabbits' antibodies purchased from CST, American.

### 2.2. Animal Model

All experimental procedures are in accordance with the animal welfare law of the People's Republic of China (ethics approval number—SCXK (Beijing) 2012-0001). Male Sprague–Dawley rats were purchased from Beijing Weitong Lihua Experimental Animal Technology Co., Ltd. The rat model of MI was replicated by ligation of the anterior descending branch of the left coronary artery. A preoperative 12-lead electrocardiogram examination was performed. After the rats were weighed, 1% pentobarbital sodium (50 mg/kg) was intraperitoneally injected for anesthesia. About 2 mm ligation of the left coronary artery was performed at the bifurcation of the coronary artery under the left auricle, and immediately the myocardial tissue below the ligation site became pale. The sham control group underwent parallel surgery, but only threading, without ligation. Two 12-lead electrocardiograms (ECG) were recorded after the operation, and the ST segment and pathological Q wave of the two electrocardiograms were compared to determine whether the modeling operation was successful or not. All animals were given penicillin intraperitoneally for three days after surgery to prevent infection. The rats were randomly divided into the model group, the Carvedilol group (1.125 mg/kg/d), and the Wenxin granules group (1.35 g/kg/d). Meanwhile, sham rats were used as the control group. 10 rats were allocated in each group. All the above drugs were dissolved in deionized water and administered by gavage once a day for 4 weeks. The model group and the control group were given deionized water.

### 2.3. Hemodynamic Detection Method

The changes in cardiac hemodynamics in each group were measured by the catheter after 4 weeks of treatment. The pressure transducer was connected to the bl-420f biological function experiment system, and then rats were anesthetized by intraperitoneal injection with 1% pentobarbital sodium (50 mg/kg), and the right common carotid artery was separated. Next, the epidural catheter is inserted through an incision in the artery wall and pushed slowly toward the heart. During the operation, the appropriate amount of heparin saline was pushed into the three channels according to the needs. At the same time, the pressure curve was watched closely. When diastolic blood pressure suddenly drops to around 0 mmHg, it indicates that the catheter has entered the left ventricle. At this point, the catheter is fixed, and a stable left ventricular pressure curve is recorded. Main test parameters include left ventricular systolic pressure (LVSP), left ventricular end-diastolic pressure (LVEDP), the maximum rate of left ventricular pressure rising (+d*p*/d*t*_max_), and the maximum rate of left ventricular pressure falling (−d*p*/d*t*_max_).

### 2.4. HE Staining Method

Paraffin sections of rat myocardial tissue were dewaxed by xylene for 3 times, each time for 15 min. 100%, 100%, 95%, 90%, 80%, and 70% ethanol was used to hydrate the sections, and nontoxic and environmentally friendly hematoxylin-eosin (HE) stain was used. The sections were stained with Hematoxylin dye for 5 min, washed with water for 3–5 seconds, Dyed with eosin dye solution for 30 seconds, rinsed with toner for 1-2 times, and dryed with filter paper or air. Coverslip was sealed with the neutral mounting medium.

### 2.5. Masson Collagen Staining (Aniline Blue) Method

Paraffin sections of rat myocardial tissue were dewaxed by xylene for 3 times, each time for 15 min. 100%, 100%, 95%, 90%, 80%, and 70% ethanol was used to hydrate the sections. Wet the glass slides with deionized water for 30–60 seconds before staining. The reagent R1 nuclear dye was used for dyeing for about 60 seconds and then was poured and rinsed for about 30 seconds with flushing fluid. Use reagent R2 pulp dyeing solution to dye for about 30–60 seconds, throw it away, and rinse with flushing solution for about 30 seconds. Reagent R3 (yellow color separation solution) was used for color separation for about 6–8 min, and the color separation solution was discarded. Dye directly with R4 (blue complex solution) for about 5 min; then, pour it out and flush with running water. 70%, 80%, 90%, 95%, 100%, and 100% ethanol was used to dehydrate the sections. Place the sections three times in xylene solution for 10 minute each. Then, the coverslips were sealed with the neutral mounting medium. Staining results show that the collagen fibers were blue, the cytoplasm is red, and the nucleus is blue-purple.

### 2.6. Immunohistochemical Staining Method for Collagen Type I and Type III

Paraffin sections of rat myocardial tissue were dewaxed by xylene for 3 times, each time for 15 min. 100%, 100%, 95%, 90%, 80%, and 70% ethanol was used to hydrate the sections. Antigen repair was performed with pH 6.0 citrate and 98°C heat preservation for 10 min. After natural cooling, it was transferred to the humidor box and soaked in PBS buffer for 5 min. Add appropriate amount of 3% H_2_O_2_ deionized water and incubate at room temperature with avoiding light for 10 min to block endogenous peroxidase. Then, wash it by PBS buffer 3 times, each time for 3 min. Drop appropriate amount of the first antibody working fluid which is diluted proportionately on the section and incubate overnight at 4°C. The next day, wash it by PBS buffer 3 times again, each time for 3 min. The appropriate amount of enzyme-labeled goat anti-rabbit IgG polymer was added and incubated at room temperature for 20 min. Then, wash it by PBS buffer 3 times, each time for 3 min. DAB color rendering was done at room temperature for 3 min. 70%, 80%, 90%, 95%, and 100% ethanol was used to dehydrate the sections. Place the sections three times in xylene solution for 10 minute each. The coverslip was sealed with the neutral mounting medium.

### 2.7. Western Blotting Method

Four weeks after treatment, the experimental rats were anesthetized by an intraperitoneal injection of 1% pentobarbital sodium (50 mg/kg) and then put to death. The cardiac tissue was isolated and stored in a liquid nitrogen environment to be measured. The tissues were removed from the liquid nitrogen and put into a precooled mortar for rapid grinding. Then, the powder was loaded into precooled EP tubes, and 50 *μ*l of cell lysate was added to each tube and put on ice for 20 min. Then, it was centrifuged at 4°C and 1000 r/min for 10 min. After centrifugation, the supernatant was removed to another precooled EP tube, and the precipitation was discarded. The protein content was determined by the BCA method, the remaining protein samples were added to 2 × SDS sample loading buffer with equal volume and boiled for 5 min. 10% SDS-PAGE separation gel was prepared, and the protein samples were separated by 10% SDS-PAGE and transferred to cellulose nitrate membrane. Then, the first antibody working solution which is diluted proportionately was added and incubated overnight under 4°C condition. Then, wash the film 3 times with tris buffer (TBS) and Tween 20 (TBST). Then, the PVDF membrane was transferred to another new hybrid bag, and the horseradish peroxidase-labeled secondary antibody was added, which was diluted with bleaching fluid, and incubated at room temperature for 2 hours, with ECL luminescence, rinsed with water, and dried for preservation. After scanning, ImageJ software was used to analyze the gray value. The expression intensity of the target protein was normalized by internal reference. Finally, the relative expression level of the target protein was determined.

### 2.8. Statistical Methods

SPSS software package version 13.0 statistical software was used for statistical analysis, and the experimental data were represented by mean ± standard deviation ($\bar{x}+s$). First, the normal distribution test and variance homogeneity tests were carried out. The continuous variables conforming to the normal distribution were statistically analyzed by one-way ANOVA. Minimum significant difference (LSD) method was used to compare the mean of multiple groups when the homogeneity of variance test was passed, and Tamhane's T2 method was used to compare the mean of multiple groups when the homogeneity of variance test was not passed. A nonparametric test was used for statistical analysis of data that did not conform to normal distribution. All the above significant levels were *P* < 0.05, indicating statistically significant differences.

## 3. Results

### 3.1. Evidence of Successful LAD Ligation in Rats

**Figure 1:**
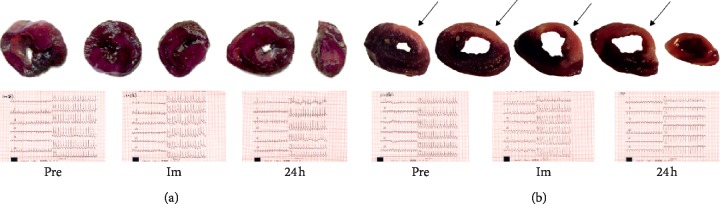
12-lead electrocardiogram and NBT staining. (a) No myocardial infarction was observed in the heart without LAD ligation. (b) The position indicated by the arrow is the infarcted area. ST segment elevation and pathological Q wave were observed in the heart after LAD ligation. Note that myocardial tissue of infarction is pale and unstained, whereas the normal myocardium is dark purple.

The myocardial infarction model in rats was prepared by LAD ligation. During the operation, the myocardial tissue below the ligation site became pale due to ischemia immediately after ligation. Immediately after ligation, 12-lead electrocardiogram examination showed that ST segment was significantly elevated. On the second day, 12-lead electrocardiogram examination was performed again, and the appearance of pathological Q wave could be observed. NBT (nitroblue tetrazolium) staining showed massive myocardial infarction after coronary ligation (Figure 1). All the above mentioned proved that myocardial infarction was successfully prepared by LAD ligation, and the survival rate of rats after LAD ligation was up to 80%.

### 3.2. Effects of Wenxin Granules on Hemodynamics of Myocardial Infarction Rats

**Figure 2:**
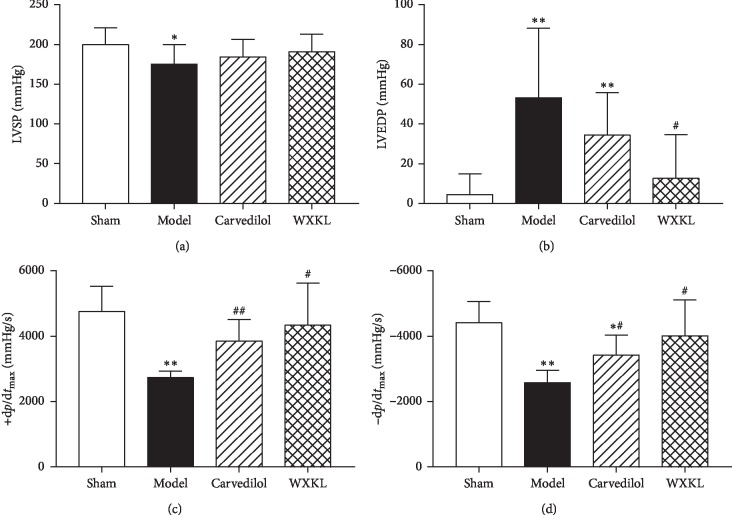
Changes of cardiac hemodynamics in each group of rats (*n* = 10). Compared with the sham group, ^*∗*^*P* < 0.05 and ^*∗∗*^*P* < 0.01. Compared with the model group, ^#^*P* < 0.05 and ^##^*P* < 0.01. Comparison of (a) LVSP, (b) LVEDP, (c) +d*p*/d*t*_max_, and (d) −d*p*/d*t*_max_ in each group.

Hemodynamics is an important indicator of heart function. The structure of matter determines the function of matter, so hemodynamics can also indirectly indicate whether the structure of the heart has been changed. The experimental results showed that, compared with the sham group, LVSP and +d*p*/d*t*_max_ and −d*p*/d*t*_max_ in the model group were significantly reduced, and the LVEDP was significantly increased. This result fully indicates that the systolic and diastolic function of the heart is greatly impaired after MI, and it can be inferred that the tissue structure of the heart must be changed, which is consistent with the clinical features of MI. Compared with the model group, there was no significant difference in the LVSP and the LVEDP between the carvedilol group, while +d*p*/d*t*_max_ and −d*p*/d*t*_max_ were significantly increased, and there was no significant difference in LVSP between the Wenxin granules group, but the LVEDP was relatively lower, while +d*p*/d*t*_max_ and −d*p*/d*t*_max_ were also significantly reduced (Figure 2). This suggests that the drug improves the systolic and diastolic function of the left ventricle after MI, and the mechanism may be to change the structural properties of myocardial tissue in the left ventricle.

### 3.3. Effects of Wenxin Granules on Myocardial Histopathology in Myocardial Infarction Rats

**Figure 3:**
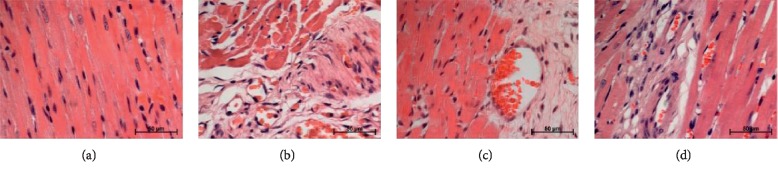
Pathological changes of left ventricular myocardium in each group of rats. The nuclei are dyed blue and the cytoplasm red. (a) The sham group. (b) The model group. (c) The carvedilol group. (d) The Wenxin granules group.

Pathological sections are the most direct observation experiments to verify the changes in cardiac tissue structure. In the sham group, the nuclei of myocardium cells were clear, and the nuclear membrane and nucleoli were visible. Cytoplasmic staining was uniform, muscle fibers were neatly arranged, and tissues were dense. But in the model group, large areas of the fibrotic scar can be seen in the infarct junction, with uneven internal density, some dense and some loose scar tissue. The residual myocardial fibers were disordered, the intercellular space was widened, and the membrane of some residual myocardial cells was blurred. This result demonstrated that in MI rats, after myocardial ischemia injury, a large number of working cells were lost, and the vacant area was replaced by scar formed by collagen deposition, whose contractility was much lower than that of myocardial working cells. A moderate amount of scar is the repair of the myocardium, so it will affect the systolic and diastolic function of the myocardium to varying degrees; so, hypertrophic scar caused by excessive collagen deposition will definitely seriously affect cardiac function. Further observation showed that in the Carvedilol group and the Wenxin granules group, fibrosis scar can be seen in the infarct junction area, and the scar tissue is relatively dense and uniform. The residual myocardial fibers were arranged neatly, and the intercellular space was slightly widened (Figure 3). This suggests that the drug improves the structural properties of the scar and residual myocardial tissue.

### 3.4. Effects of Wenxin Granules on Collagen Volume of Myocardial Tissue in Myocardial Infarction Rats

**Figure 4:**
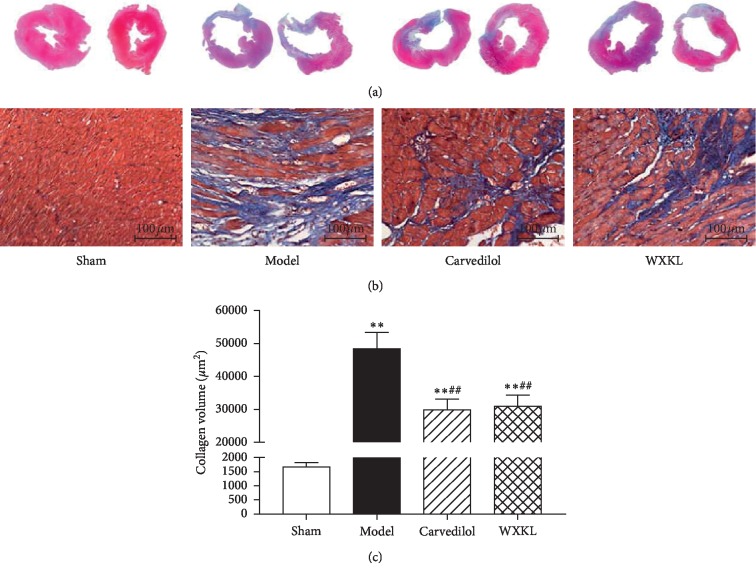
Changes of collagenous volume in the left ventricular myocardium in each group (*n* = 10). Compared with the sham group, ^*∗*^*P* < 0.05 and ^*∗∗*^*P* < 0.01. Compared with the model group, ^#^*P* < 0.05 and ^##^*P* < 0.01. (a) Masson staining images at low magnification, one group in two, from left to right, respectively, the sham group, the model group, the carvedilol group, the Wenxin granules group. (b) Masson staining images at high magnification of each group. (c) Statistics of volume integral of collagen in each group.

In the disease course of heart failure after MI, the passive stiffness of the overall myocardium becomes collagen dependent, which is closely related to the collagen content and shows that the proinflammatory and profibrosis signal plays an important role in this process [12]. In this study, the changes in collagen volume after MI in rats were observed by Masson staining. The results showed that there was no significant myocardial infarction in the sham group, while the myocardium in the model group at the ligation site was thinner and mainly composed of collagen, while the myocardium in the carvedilol group and the Wenxin granules group was relatively improved (Figure 4(a)). The results also showed that the sham group had uniform staining, neat arrangement, dense tissue, and only a small amount of collagen expression between myocardial cells. In the model group, large areas of fibrotic scar stained blue were seen in the infarct junction area, and the internal density of scar tissue was uneven, some dense, and some loose. The remaining cardiomyocytes are divided and surrounded by collagenous fibers, which are arranged in disorder. The intercellular space is widened and filled with numerous proliferating collagenous fibers. The infarct junction of the Carvedilol group and the Wenxin Granules group showed fibrosis scar stained blue, and scar tissue was relatively dense and uniform. Residual myocardial cell gap widened slightly, which can be seen in the clearance of collagen fiber hyperplasia (Figure 4(b)). Image analysis was carried out on the dyeing results, the results show that, compared with the sham group, the collagen volume of myocardial tissue in the model group, the Carvedilol group, and the Wenxin Granules group was significantly increased, but compared with model group, the collagen volume of the Carvedilol group and the Wenxin Granules group was significantly decreased (Figure 4(c)). This suggests that excessive deposition of collagen did occur in the myocardial mesenchyme of MI rats, which is consistent with the inference that myocardial stiffness increased and function decreased, and the drug did play a role in reducing collagen deposition, suggesting that the drug may improve the structural properties of tissues by affecting collagen deposition.

### 3.5. Effects of Wenxin Granules on the Expression Area of Type I and Type III Collagen in Left Ventricular Myocardium of Myocardial Infarction Rats

**Figure 5:**
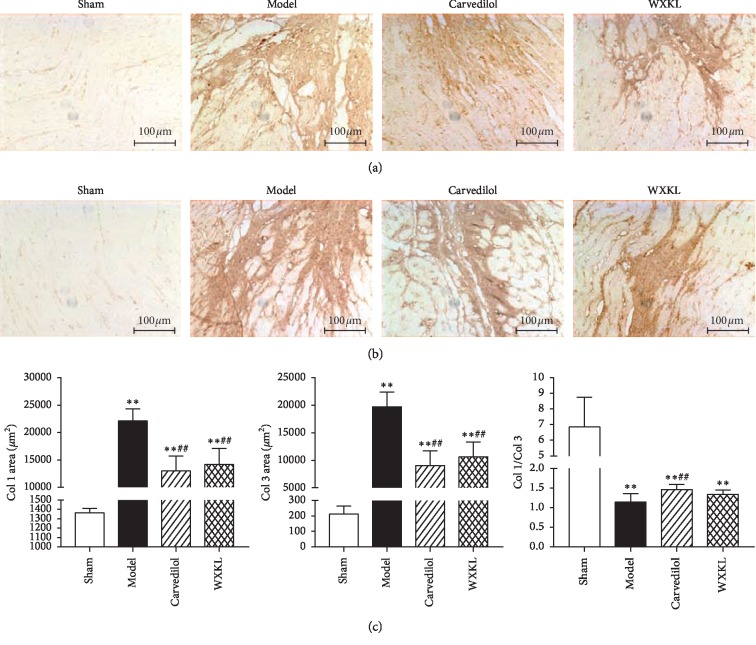
Changes of type I and type III collagen expression area in myocardial tissue of the left ventricle in each group of rats (*n* = 10). Compared with the sham group, ^*∗*^*P* < 0.05 and ^*∗∗*^*P* < 0.01. Compared with the model group, ^#^*P* < 0.05 and ^##^*P* < 0.01. (a) Results of type I collagen immunohistochemical staining, from left to right, were the sham group, the model group, the carvedilol group, and the Wenxin granules group. (b) Results of type III collagen immunohistochemical staining, from left to right, were the sham group, the model group, the carvedilol group, and the Wenxin granules group. (c) Collagen expression area statistics, from left to right, were Col I, Col III, and Col I/Col III.

The main component of ECM is collagen, and collagen I and III is specifically expressed in the myocardium [6]. Immunohistochemistry stain of type I and type III collagen was performed in this study. The results showed that the majority of the areas in the sham group were in pale-yellow background color, during which only a small amount of brown and yellow thin filament-type collagen fibers could be observed, and no staining blank caused by tissue loss was observed. However, in the model group, large areas of brown-yellow type I and type III collagen fibers were found in the infarct junction area, showing large patchy patches with uneven internal coloring, and a large number of blank areas formed by tissue loss were found in or around the patch. The residual myocardium showed a yellowish background color, during which there were a large number of irregular brown and yellow cable-like collagen fibers expressed (Figure 5(a)).

Compared with the sham group, the area of collagen expression in the model group, the Carvedilol group, and the Wenxin granule group increased significantly. Compared with the model group, the expression area of type I and type III collagen in the Carvedilol group and Wenxin granule group was significantly reduced. In the infarct junction area of the Carvedilol group and the Wenxin Granules group, the expression of type I and type III collagen fibers dyed brown and yellow in a certain area was observed, which was patchily stained, and the internal coloration of the patchily was relatively uniform. The residual myocardium was yellowish in the background, and a small amount of brown-yellow filamentous collagen fibers were observed (Figure 5(b)). Based on the above experiments, this part successfully further verified that the main changes of ECM in MI rats were characterized by collagen deposition, while the drug could reduce it.

In addition to changes in the collagen expression area, the ratio of different types of collagen also has an impact on cardiac function. Collagen type I and type III are the most expressed collagen types in the heart. Under physiological conditions, collagen type I in the heart accounts for about 80% of the total amount of collagen, with strong extensibility. Collagen type III accounts for about 10% and has a good recovery. The two types of collagen maintain a certain ratio and together maintain normal systolic and diastolic function [13]. The imbalance of this ratio of different types of collagen would lead to increased tissue stiffness and decreased compliance. In this experiment, we found that compared with the control group, the ratio of type I collagen to type III collagen in the model group and the Carvedilol group and the Wenxin Granules group decreased significantly. But compared with the model group, the ratio of type I collagen to type III collagen in the Carvedilol group was obviously higher, and the Wenxin granules group had a higher trend, but no statistical difference (Figure 5(c)). This indicates that the drug reduces the deposition of collagen very well, but has a limited effect on the improvement of adjusting the proportion of collagen.

### 3.6. Effects of Wenxin Granules on the TGF*β*-P38/JNK MAPK Signaling Pathway

**Figure 6:**
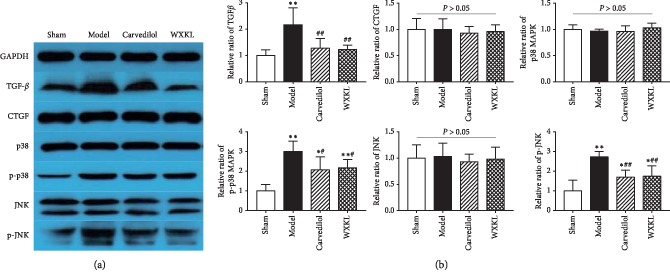
Changes in TGF*β*-p38/JNK MAPK signaling pathway (*n* = 4). Compared with the sham group, ^*∗*^*P* < 0.05 and ^*∗∗*^*P* < 0.01. Compared with the model group, ^#^*P* < 0.05 and ^##^*P* < 0.01. (a) Expression of target protein in each group. (b) The relative expression statistics of target protein in each group, the bar chart shows, from left to right, the sham group, the model group, the carvedilol group, and the Wenxin granules group.

As we all know, TGF*β* is considered to be a major cytokine/growth factor produced in damaged or diseased tissues. It is a major inducer of matrix chemokines and has a significant and far-reaching impact on ECM components and inflammatory cytokines [14]. Generally, TGF*β* is highly expressed in myocardial injury, while CTGF has synergistic effects with it [15]. These two key factors bind to cell surface receptors and activate a series of intracellular signals that promote the synthesis of ECM components such as collagen, fibronectin, and elastin [16]. Using the WB experiment of semiquantitative analysis target protein in the organization (Figure 6(a)) and compared with the control group, the model group had a significantly higher relative expression of TGF*β*, but relative expression of TGF*β* in the Carvedilol group and the Wenxin granules group only increased and no statistical difference. Compared with the model group, the relative expression of TGF*β* in the Carvedilol group and the Wenxin granules group were significantly lower. No statistical difference was found in the relative expression level of CTGF between groups. This indicates that in this experimental model, CTGF was not activated by ischemia stimulation and obviously did not exert a synergistic effect. Therefore, compared with the results of previous experiments, TGF*β* is a more dominant inflammatory stimulus generated in the myocardial mesenchyme after ischemia. In this study, it can be inferred that TGF*β* is a key factor affecting collagen deposition.

TGF*β* can initiate noncanonical signal transduction and activate MAPK cascade, eventually activating p38, JNK1/2 signaling pathways [17]. MAPK signaling cascade is one of the specific intracellular processes that recognize and respond to extracellular stimuli. All eukaryotic cells have multiple MAPK pathways regulating the whole intracellular activities, such as inflammation, apoptosis, differentiation, and so on. MAPKs can be activated by multiple stimuli, but generally, p38 and JNK signaling pathways are preferentially activated [18]. The experimental results showed that there was no statistical difference in the relative expression of P38 between the groups. However, the relative expression level of p-p38 showed significant differences among groups, suggesting that the phosphorylation level of p38 protein changed greatly. Compared with the sham group, the relative expression level of p-p38 in the model group increased significantly, and the same phenomenon also occurred in the expression of JNK protein. This indicates that TGF*β*, an inflammatory stimulant after MI, is an upstream activator of the p38/JNK MAPK signaling pathway. TGF*β* may affect the inflammatory response and collagen expression by initiating the p38/JNK MAPK signaling cascade.

Compared with the model group, p-p38 and p-JNK relative expression in the Carvedilol group and the Wenxin Granules group were significantly lower (Figure 6(b)); this directly verifies the drug acting on the p38/JNK MAPK signal cascade. According to the result of all the above experiments, TGF*β* can activate the p38/JNK MAPK pathway and affects collagen deposition, while Carvedilol and Wenxin granules can change the phosphorylation level of key proteins in this pathway to limit inflammatory response and reduce excessive deposition of collagen.

## 4. Discussion

In the transitional state from the inflammatory stage to the repair stage after the occurrence of MI, infiltrating inflammatory cells and matrix binding substances in ECM activate TGF*β* and other factors, trigger intracellular related effects and downstream signaling cascade, and play an important role in regulating the inflammation and wound healing as well as tissue remodeling [19]. Under the stimulation of inflammation after MI, the pathological changes of ECM are characterized by excessive accumulation of major components and the transition to repair fibrosis, which means collagen deposition scar instead of injured myocardium [20]. Collagen deposition is the key mechanism of myocardial fibrosis and has a direct impact on cardiac function [21]. Previously, in a study of clinical heart failure patients, left endocardial myocardial biopsy quantified the ECM region and tested the effect of ECM on invasive hemodynamic parameters [22]; it is proved that hemodynamic changes are directly related to the deposition degree of ECM. In this experiment, catheterization was used to detect hemodynamics in each group of rats, and the results were consistent with the above experimental conclusions. The role of the inflammatory response in disrupting the balance of collagen metabolism after MI has also been preliminarily verified in this study. In the inflammatory response, the production of TGF*β* decreases, while the deposition of collagen in ECM increases, and the p38/JNK MAPK signal cascade also changes in the corresponding trend. The metabolic balance of collagen is necessarily the balance of synthesis and degradation. Whether the inflammatory pathway directly and specifically affects the synthesis and degradation of collagen is a question worthy of further exploration. Some studies suggest that promoting the apoptosis of interstitial cells and degradation of interstitial components is one of the effective means to reduce and reverse fibrosis. Among them, the phosphorylation level of P38/JNK was increased by increasing intracellular calcium concentration that may be one of the mechanisms targeting apoptotic mesenchymal cells [23]. Despite the contrary views, the changing trend of the P38/JNK phosphorylation level in fibrosis response was consistent. Combined with the results of this experiment, this paper is more inclined to support the view of inhibiting the P38/JNK MAPK signaling pathway, downregulating TGF*β*, and inhibiting the phosphorylation level of P38/JNK MAPK. It may even limit the extent of collagen deposition and promote the benign remodeling of the ventricle. Currently, there are no effective means to inhibit or reverse the ECM lesions after ischemia, and the intervention of collagen metabolism may be a useful approach to preliminarily clarify the mechanism of the intervention of inflammation to influence collagen deposition, which may open up new prospects for improving ventricular remodeling after MI.

The ultimate purpose of studying the effect of inflammation on collagen is to study the effect of inflammation on myocardial fibrosis. Collagen deposition is the key mechanism of myocardial fibrosis. TCM has played an unexpected role in the intervention of fibrosis in various organs. No matter it is a TCM compound or TCM monomer or its component, it can significantly inhibit or weaken fibrosis in organs [24–28]. The effect of natural drug components on fibrosis has always been a research hotspot in the field of TCM. For example, astragalus polysaccharide, astragaloside IV, cycloastragalus alcohol, total astragalus saponins, and other effective ingredients can interfere with pulmonary fibrosis. Panchiopsis codonopsis can effectively delay the decline of glomerular filtration rate in stage 2 to 3 of chronic kidney disease, improve clinical symptoms such as anemia, and slow down the process of renal interstitial fibrosis. Radix codonopsis polysaccharide has also been shown to inhibit ischemia-reperfusion injury [29, 30]. Although TCM has shown great clinical effects, there is still a lack of research on the upstream influencing factors of TCM on fibrosis. Fibrosis is, to some extent, a pathological end-result of inflammatory response. Currently, there are few studies on the intervention of TCM on myocardial fibrosis, but the occurrence and development of myocardial fibrosis and its significance on the prognosis of cardiovascular diseases have become the consensus of the medical community. As a consequence, it is necessary to draw lessons from TCM of natural medicine or compound study of other tissue fibrosis intervention, seeking possible effective intervention on myocardial fibrosis drug of TCM. Meanwhile, the specific mechanism of action and the influence of upstream factors such as inflammation on fibrosis should be studied.

Based on this, this study took Carvedilol as the positive control drug in the experiment to study the effect of Wenxin granules on the inflammatory response after MI and the myocardial fibrosis response mainly manifested by collagen deposition. Carvedilol is a beta-blocker, and there is ample evidence that Carvedilol has a beneficial effect on left ventricular systolic dysfunction (LVSD) after MI [31] and gives priority to improving the REDOX status of the heart and can prevent cardiac dysfunction and REDOX imbalance after MI [32]. The latest randomized controlled double-blind clinical trial also demonstrated that Carvedilol significantly reduced cardiac troponin levels and diastolic dysfunction [33] and reduced biomarkers of tissue fibrosis and inflammation in hypertensive disease models, such as TGF*β* [34]. Therefore, using Carvedilol as positive control can ensure the effectiveness of the Wenxin granule compound in the experiment. The components of the Wenxin granule compounds are codonopsis, *Polygonatum sibiricum*, *Panax notoginseng*, amber, and *Nardostachys chinensis*, which contain a large number of beneficial components that interfere with tissue inflammation and fibrosis. In many previous experiments, Wenxin granules have been not only verified to have a significant impact on cardiac electrophysiological remodeling, but also to participate in ventricular remodeling after myocardial injury by regulating many systems and markers, such as oxidative stress, inflammatory response, fibrosis, neuroendocrine regulatory factors, and vascular endothelium-related factors. The experimental results of this study demonstrated that Wenxin granules can attenuate collagen deposition and change the structural properties of the scar so as to improve systolic function and improve the function of residual myocardium by increasing adaptive injury of cardiomyocytes. It also downregulates the expression of inflammatory stimulant TGF*β* and inhibits the phosphorylation response of the p38/JNK signaling cascade.

In conclusion, Wenxin granules can significantly improve the hemodynamics of MI rats and change the material properties of scar structure and residual myocardial structure, reduce the expression of type I and III collagen and increase the ratio of type I to type III collagen, and downregulate inflammatory stimulus signal TGF*β* and reduce the phosphorylation level of p38/JNK. It can be considered that the Wenxin granules can regulate the inflammation-related TGF*β*-p38/JNK MAPK signaling pathway and reduce excessive collagen deposition and play a protective role in cardiac function after MI by improving the structural properties of myocardial tissue.

## 5. Conclusion

Wenxin granules may regulate the inflammation-related TGF*β*-p38/JNK MAPK signaling pathway and attenuate the collagen deposition of ECM, so as to inhibit the damage of myocardial tissue structure caused by ischemia injury after MI and improve cardiac function by changing the structural properties of myocardium and scar.

## Data Availability

The data used to support the findings of this study are available from the corresponding author upon request.
